# Interactions between Parents and Parents and Pups in the Monogamous California Mouse (*Peromyscus californicus*)

**DOI:** 10.1371/journal.pone.0075725

**Published:** 2013-09-19

**Authors:** Cheryl S. Rosenfeld, Sarah A. Johnson, Mark R. Ellersieck, R. Michael Roberts

**Affiliations:** 1 Bond Life Sciences Center, University of Missouri, Columbia, Missouri, United States of America; 2 Department of Biomedical Sciences, University of Missouri, Columbia, Missouri, United States of America; 3 Agriculture Experimental Station-Statistics, University of Missouri, Columbia, Missouri, United States of America; 4 Department of Animal Sciences, University of Missouri, Columbia, Missouri, United States of America; 5 Department of Biochemistry, University of Missouri, Columbia, Missouri, United States of America; Michigan State University, United States of America

## Abstract

The California mouse ( *Peromyscus* *californicus* ) may be a valuable animal model to study parenting as it is one of the few monogamous and biparental rodent species. By using automated infra-red imaging and video documentation of established pairs spanning two days prior to birth of the litter until d 5 of post natal development (PND), it was possible to follow interactions between parents and between parents and pups. The paired males were attentive to their partners in the form of grooming and sniffing throughout the time period studied. Both these and other activities of the partners, such as eating and drinking, peaked during late light/ mid-dark period. Beginning the day before birth, and most significantly on PND 0, the female made aggressive attempts to exclude the male from nest-attending, acts that were not reciprocated by the male, although he made repeated attempts to mate his partner during that period. By PND 1, males were permitted to return to the nest, where they initiated grooming, licking, and huddling over the litter, although time spent by the male on parental care was still less than that of the female. Male and female pups were of similar size and grew at the same rate. Pups, which are believed to be exothermic for at least the first two weeks post-natally, maintained a body temperature higher than that of their parents until PND 16. Data are consistent with the inference that the male California mouse parent is important in helping retain pup body heat and permit dams increased time to procure food to accommodate her increased energy needs for lactation. These assessments provide indices that may be used to assess the effects of extrinsic factors, such as endocrine disrupting chemicals, on biparental behaviors and offspring development.

## Introduction

The influence of early maternal care on offspring outcomes is receiving considerable attention. It is clear from several studies that the quality and quantity of maternal care can lead to dramatic consequences in later life, including morphological changes in the brain and effects on later play, anxiety, fear, and reproductive behaviors [1,2,3,4,5,6,7,8]. These changes are likely a result of epigenetic imprints placed on key genes during the time when maternal care is most critical to the future well-being of the progeny [9,10,11,12,13,14,15,16]. The impact of paternal care has been less studied and is therefore less well understood, probably because relatively scant number of mammalian species exhibit both monogamy and biparental care and of those that do few of those provide useful experimental models for studying offspring emotional, social, and cognitive development [17,18].

Rodents, because of their small size and short reproductive cycles, have proven to be ideal models in biomedical studies, but only a few rodent species are monogamous and exhibit well defined patterns of bipaternal care [19,20,21]. Examples include several species from the genus *Peromyscus* , notably the California mouse ( *P* *. californicus* ), the oldfield or beach mouse ( *P* *polionotus* ), and the cactus mouse ( *P* *. eremicus* ). Additionally, some vole species are also monogamous and biparental, including prairie voles ( *Microtus* *ochrogaster* ), pine voles ( *M* *. pinetorum* ), and mandarin voles ( *Lasiopodomys* *mandarinus* ). Within the *Peromyscus* and *Microtus* genera, there are also polygynous species such as deer mice ( *Peromyscus* *maniculatus* *bairdii*) and meadow voles ( *Microtus* *pennsylvanicus* ), respectively, where the male plays no role in raising the young and his presence may even decrease pregnancy success [22,23,24]. Males of most inbred and outbred strains of laboratory mouse, *Mus musculus*, do not appear to demonstrate much involvement in pup rearing [25]. Of the various species, California mice, prairie voles, and mandarin voles have been most widely used to study this aspect of reproduction.

California mice inhabit environments ranging from mountainous to near sea level. The height of their activity occurs within a few hours of nightfall and predawn [26]. Details of paternal behavior in this species were first reported in 1935 [27]. Trapping data revealed a high rate of nest site fidelity and long term association of the paired male and female [28], features of behavior consistent with monogamy and biparental care. Laboratory studies have confirmed lasting pair bonds [29,30]. The presence of the father has been inferred to accelerate the growth and development of the offspring [31,32]. Additional studies have supported the notion that California mice evolved biparental care in response to resource availability. Not only may biparental investment prove beneficial to the health and well-being of offspring, it also appears to promote development of the hippocampus [33,34,35], elicit hormonal changes in the pups [36], and enhance certain forms of future adult behavior, such as aggression in either sex [37]. Finally, monogamy accompanied by paternal investment has been inferred to permit maternal behaviors to evolve cooperatively within the biparental circumstance [22]. Consequently, the California mouse provides an excellent opportunity to investigate the ways in which the parents work together to rear socially competitive offspring [22,34,38].

To our knowledge, only one prior study has performed comprehensive video documentation of the interrelationship between the sexes after birth in California mice [39]. In that work, a time-lapse video cassette recorder was used to record activities during the light period, while dark activities were tracked in separate cages under red light illumination [39]. However, the design of the study precluded data gathering on the same pairs of mice through the entire light/dark cycle. The study also did not follow pair-bond social behaviors prior to and in the hours immediately after birth. The recent development of cages equipped with automated infra-red cameras now permits analyses to be made from archival videos taken from above the cage for fixed periods at any preselected stages of the light/dark cycle, including during the birth period. Individual parental behaviors can also be followed without having to remove one parent from the cage, a practice commonly employed in most previous studies [31,32,34,40,41,42].

Here, we have used such an infra-red, continuous monitoring to assess some of the complex interactions that occur between the parents and between the parents and their offspring in the California mouse during the period immediately prior to and after birth of the pups. Additionally, we have measured pup weight and body temperature as indices of outcomes of normal biparental care, as it has been postulated that in *P* *. californicus* , care of the offspring by the males permits the female increased time outside of the nest to forage for food [31,32], during which time the male huddles over the litter, most likely to prevent a major drop in body temperature in the pups, which appear to be markedly exothermic until about PND 15 [39]. The hypothesis prompting these studies was that these monogamous and biparental animals would demonstrate key pair-bonded behaviors and shared parenting responsibilities that might be vulnerable to extrinsic factors. Our longer term goals, therefore, are to examine how such factors, especially endocrine disrupting chemicals in the diet, influence this suite of behaviors and consequent development of the young.

## Materials and Methods

### Animal husbandry

The original founder outbred adult (60-90 days of age) founder California mouse females and males, free of common rodent pathogens, were purchased in 2010 from the *Peromyscus* Genetic Stock Center (PGSC) at the University of South Carolina (Columbia, SC), and placed in quarantine for a minimum of 8 weeks to ensure that they did not carry any transmittable and zoonotic diseases. From the time the animals had been captured between 1979 and 1987, *P* *. californicus* captive stocks have been bred by the PGSC to maintain their outbred status. The breeders used in these experiments are ~F_5_ descendants of these original founders purchased from this facility.

All experiments were approved by University of Missouri Animal Care and Use Committee and performed in accordance with the recommendations in the Guide for the Care and Use of Laboratory Animals of the National Institutes of Health. Virgin females, 8 to 12 wks of age, (n= 6) were randomly assigned to receive a low phytoestrogen AIN 93G diet supplemented with 7% by weight corn oil to minimize potential phytoestrogenic contamination that would otherwise be present with inclusion of soybean oil in the diet. When the animals were not in the Phenotyper™ system (detailed below), they were housed in white polypropylene cages (27.8 x 7.5 x 13 cm) and maintained on a 16: 8 h light: dark cycle (lights on at 0600 hrs CST, lights off at 2200 hrs CST).

Since California mice are monogamous, one male was paired with a single female, and the pair remained together for the duration of the study. California mice do not form a vaginal or copulatory plug, as observed in laboratory mice (*Mus musculus*). To determine if the females were gravid, they were weighed weekly, and five days prior to the predicted parturition date, the breeding pair was placed in the Phenotyper™ (Noldus Technologies, Leesburg, VA). The breeding pair and pups were kept in this cage system through five days after birth. As with the cage set-up, the animals were provided filtered water in a polypropylene water bottle. California mice typically birth one to two pups in each litter, although litters sizes up to 4 have been reported and observed in our own studies [29,43,44,45,46].

### Coding of individual, social, and parental behaviors

The Media Recorder timer program (Noldus) switched on the infra-red video-cameras to record behaviors from (MD, middle of the dark period, 1.00-2.00 h; EL, early in the light period 7.30-8.30 h; ML, middle of the light period 13.00-14.00 h; LL, late in light period, 21.00-22.00 h). To distinguish the two animals in each pair, prior to breeding, each male, under anesthesia from an intra-peritoneal (IP) injection of Avertin (250 mg/kg), was marked by an approximately 2 by 3 inch area shaved along his dorsal thoracic region. The Observer Version XI program (Noldus) was used to code the archived videos. Thus, the behavior of each animal in six bonded pairs was coded four times a day for one hour periods over eight days, thereby providing strong Power to the data analyses. Two observers coded each of the videos with inter-rater reliability > 0.9. Importantly, this program permits the experimenter to rewind, freeze, or play the videos in slow motion to ensure that each behavior is accurately captured. When a specific behavior is observed, the experimenter types a lower case letter key that corresponds to that behavior, e.g. n for entering the nest. For those behaviors where duration was measured, the operator types the appropriate capital letter when the behavior ceases, e.g. “N” to demarcate when the animal leaves the nest. The program thus permits determinations of frequency as well as duration of specific behaviors (Table S1), which were coded two days prior to birth and from post-natal day (PND) 0 (day of birth) to PND 5. This time range was chosen based on preliminary assessments that had extended out to PND 15 and revealed that the most dramatic changes in biparental behaviors were observed through PND 5. The resulting data generated from the Observer Program (Noldus) were imported into Microsoft Excel to allow statistical analyses to be performed.

### Determination of pup body weight and temperature, nest temperature, and parent body temperature

For these analyses, an additional five litters from parents on the AIN 93G diet were assessed. Beginning on PND 2, the pups were gently removed from the nest (or nipple if they were suckling), placed abdomen down on a scale (OHAUS CS200, Parsippany, NJ) that was covered with a brown paper towel, and a thermal image acquired with a FLIR i5 camera (FLIR Systems Inc., Boston, MA) with the lens 22 cm above the pup. In litters, where there was more than a single pup, individual pups on PND 2 were given a distinguishing tattoo mark on one of their paws on either the front or back legs (Fine Science Tools, Foster City, CA). Before the pups were returned to the nest, a thermal image of the nest area was also obtained to assess the temperature of the nest. Measurements were obtained every two days from PND 2 to 20 and then prior to and after weaning at 8:30, 12:30 and 16: 30 h. From the five litters, a total of 7 male and 6 female pups were analyzed. The multiple days and times of analysis for these litters provided considerable Power to the data and enabled significant differences to be detected across days and different times in the light/dark cycle, as indicated in the Results section. To determine if the pup body temperature differed from those of the dam and sire, the dam and sire temperatures were determined on a weekly basis at the same times above. All thermal images were analyzed by using the FLIR Tools software program (http://flir.com/tools/). The emissivity of fur was set at 0.98, as reported previously [47,48]. Values were adjusted to represent the average temperature from the head to the base of the tail. To calculate temperature of the nest, which was constructed of aspen shavings, the emissivity was set at 0.924 based on a prior study with various wood samples [49] and the emissivity table provided by FLIR Systems Inc.

### Statistical analyses

Male and female paired behavioral data were grouped as follows: two days prior to birth, PND 0 (day of birth), PND 1-2, and PND 3-5 for each sex. For all of the PND 0 assessments, the 1.00-2.00 h (MD) timepoint was included on this day even if the pups were not born yet to allow a full rank data set for the data analysis. The male and female behaviors in a breeding pair were analyzed together and independently to determine if any pair-bond effects were evident. The behaviors that were relevant to a given sex (as detailed in Table S1) were ranked due to heterogeneous of variance [50]. Individual, social and parental behaviors were also analyzed by using the linear statistical model that contained the effect of sex, day (two days prior to birth, PND 0, PND 1-2, and PND 3-5), time (1.00-2.00 h [MD], 7.30-8.30 h [EL], 13.00-14.00 h [ML], and 21.00-22.00 h [LL]) and all possible interactions with sex, day and time. Each breeding pair within sex was considered as the denominator of F to test sex, and pair within sex effects. Secondly, day was used as the denominator of F to test day and sex X day effects, and the residual mean square of pair within sex, day, and time of day was used as the denominator of F to test time and all possible interaction of time with sex and day. To analyze the combined parental categories, time spent in nest with pups and grooming pups, the total sum for both analyses within a pair for day and time was determined. The data were analyzed as a 3 by 4 factorial arrangement of treatment. The behaviors that were relevant to a given sex (as detailed in Table S1) were ranked due to heterogeneous of variance [50]. Next, the data were analyzed by using a split split plot in time analysis [51] and SAS version 9.2 software analyses (SAS Institute, Cary, NC). This procedure is a modification of Littell et al [51] that describes a split plot in time, which is a repeated measure analysis. The split split plot in time method takes into consideration each litter as the unit and is a repeated measures analysis within day and time within day. Unless otherwise stated, the reported data are based on Mean ± SEM per hour assessments.

For the pup weight, pup body temperature, nest temperature, and parent body temperatures, three analyses were performed. Weight was analyzed as a randomized complete block design (RCBD) in which the model contained the effects of parents (combination of dam and sire), day, sex and the interaction of day X sex. The second analysis was performed on pup temperature and nest temperature data. The linear statistical model was a RCBD and split split plot in time. The mating pair was considered the complete block. Sex was the main plot, day and sex X day was the sub plot, and time and all of the interactions of time with day and sex was the sub sub plot. The third analysis for parent body temperature was a Completely Randomized Design in which the model contains the effects of time of day and parent sex. All mean differences were determined by using Fisher’s Least Significance Difference (LSD). PROC MIXED procedure in SAS 9.2 was used to analyze all of the above data.

## Results

### Individual behaviors

Besides following behaviors involving interactions between an animal and its partner and with the pups, each animal was also tracked when it was disengaged from these activities. Even so, in the case of eating and drinking, these behaviors likely impact the ability of the partners to nourish (in the case of the female) or provide parental care to their pups.

#### Eating and drinking

**Figure 1:**
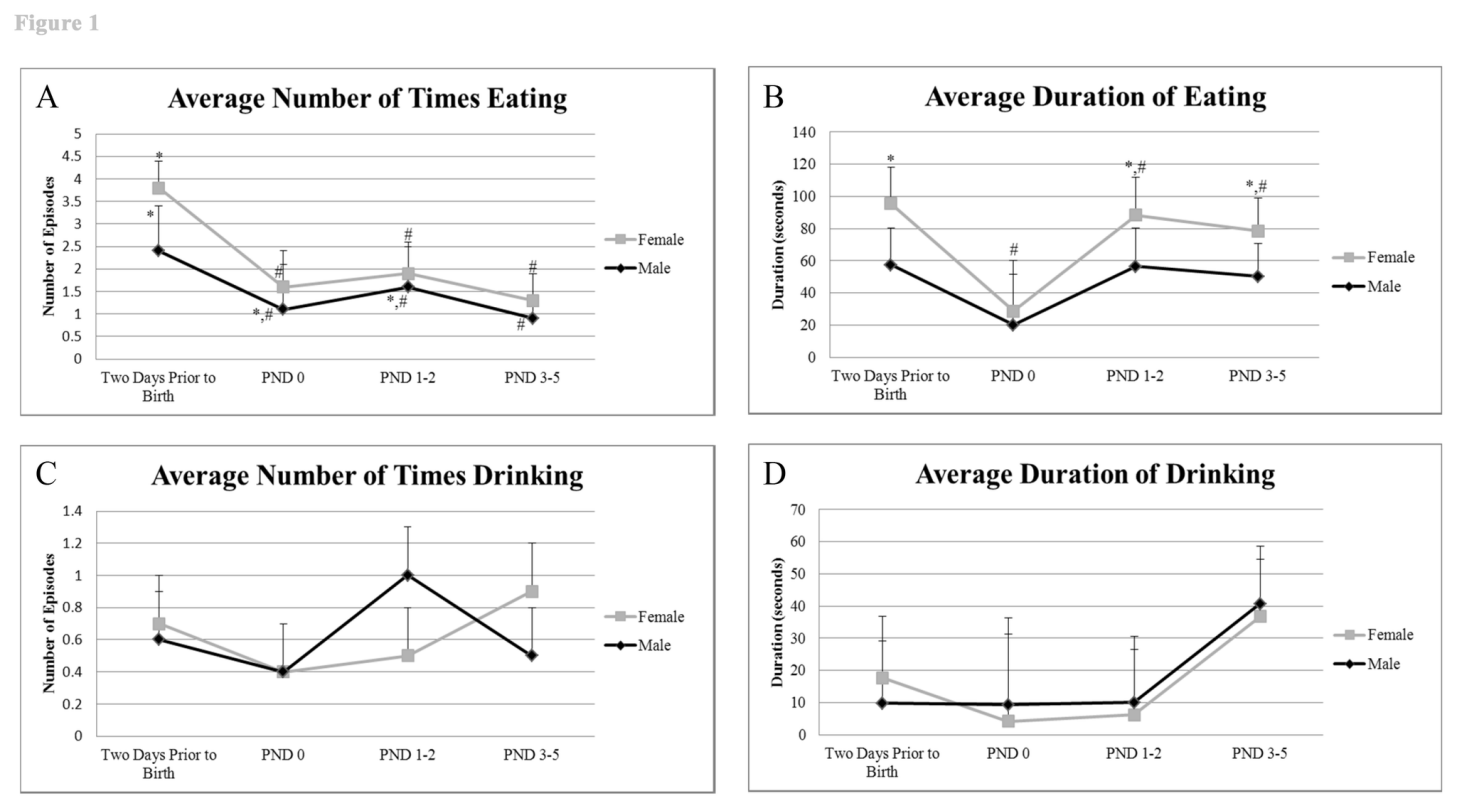
Frequency and duration of eating and drinking prior to and after birth. A) Average frequency of eating for both parents prior to and after birth. B) Average duration of eating for both parents prior to and after birth. C) Average frequency of drinking for both parents prior to and after birth. D) Average duration of drinking prior to and after birth. ^*,#^ indicates significant differences within sex across days examined (P < 0.05).

The frequency and duration of eating and drinking in both parental sexes on the selected days within the four selected time periods (MD, middle of the dark period, 1.00-2.00 h; EL, early in the light period 7.30-8.30 h; ML, middle of the light period 13.00-14.00 h; LL, late in light period, 21.00-22.00 h) were measured to assess if consumption of food and water changed after birth of pups (Figure 1). Males exhibited greater frequency of eating two days prior to birth compared to PND 3-5 (2.4 ± 0.6 versus 0.9 ± 0.6, respectively, P = 0.05), although duration of eating remained relatively unchanged (Figure 1B). No other differences between days were significant for males. Females engaged in fewer eating episodes (P value range = 0.0002 to 0.02) and these episodes were of shorter duration (P = 0.05) on the day of birth than either before or after the pups were born (Figure 1B). For example, average time spent eating by females at two days prior to birth and at PND 0, PND 1-2, and PND 3-5 was 44.0 min, 19.0 min, 40.8 min, and 37.3 min, respectively. The duration and frequency of drinking showed considerable variance, but did not vary according to day.

Predictably, the eating episodes and duration changed over the light/dark cycle (Figure S1). Both sexes were observed eating more frequently in the MD period than at other times (P value range <0.0001 to 0.0004), with the females exhibiting more eating episodes than their male partners during this period (6.9 ± 0.7 versus 4.3 ± 0.7, respectively, P= 0.01) and also spending more time eating than the males (P = 0.02). Females also drank more frequently at MD than at the other time points (P value < 0.0001); whereas, males drank longest (and presumably most) at MD and EL compared to other times (P value range 0.0008 to 0.01).

#### Self-grooming

**Figure 2:**
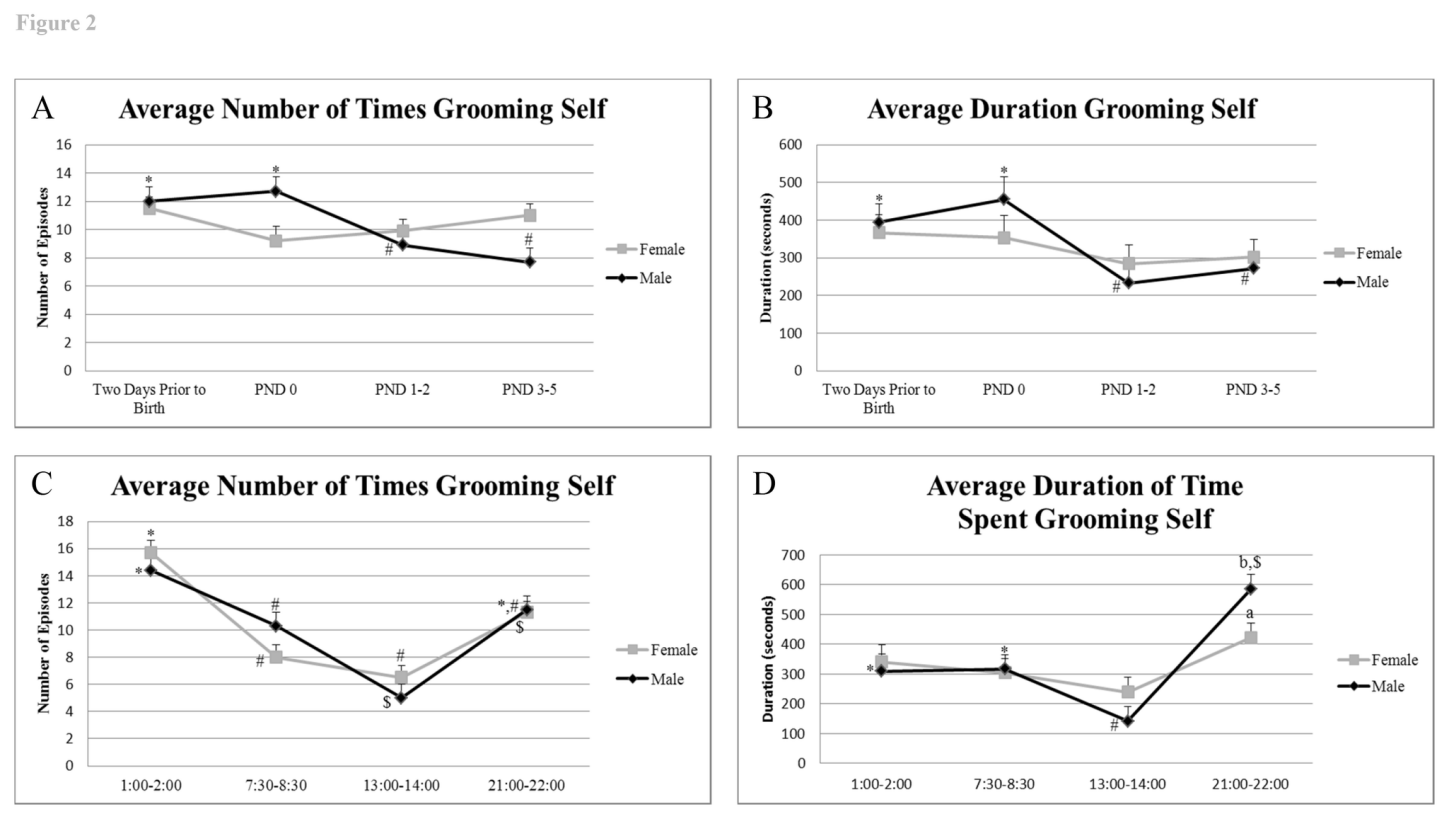
Frequency and duration of self-grooming behaviors prior to and after birth and throughout the timepoints examined. A) Average frequency of self-grooming across trial days. B) Average duration of self-grooming across trial days, C) Average frequency of self-grooming based on time of day, and D) Average duration of self-grooming based on time of day. ^*, #, $^ indicates significant differences within sex across days or times examined (P < 0.05). ^a^,^b^ indicates significant differences between sexes at the same time of day examined (P < 0.05).

Over the trial period, the males and females engaged in equal number of self-grooming episodes (P = 1.0), although, for males, their frequency was greatest prior to birth and on PND 0 than at later times (two days prior to birth= 12 ± 1.6, PND 0= 12.7 ± 1.9, PND 1-2= 8.9 ± 1.6, and PND 3-5= 7.7 ± 1.5, P value range 0.005 to 0.04) (Figure 2). Likewise, the overall duration of self-grooming did not differ between the sexes (P = 0.8). Again, males spent more time grooming prior to birth and on PND 0 than during either PND 1-2 and 3-5 (Figure 2B) (two days prior to birth= 393.6 ± 48.3 seconds, PND 0= 455.0 ± 60.4 seconds, PND 1-2= 232.5 ± 49.9 seconds, and PND 3-5= 271.6 ± 46.7 seconds, P value range = 0.0001 to 0.01). The frequency and duration of female self-grooming did not change across the days examined (Figure 2A and B).

Grooming episodes were less frequent for both sexes in the light periods (EL and ML) than in the late light/ middle dark periods (LL & MD) (P value range <0.0001 to 0.2) (Figure 2C). Males groomed themselves for longer than their partners during LL (P= 0.02) and less frequently during ML compared to the other two periods (Figure 2D) (P value range <0.0001 to 0.006).

### Social behaviors

#### Aggression to mate

**Figure 3:**
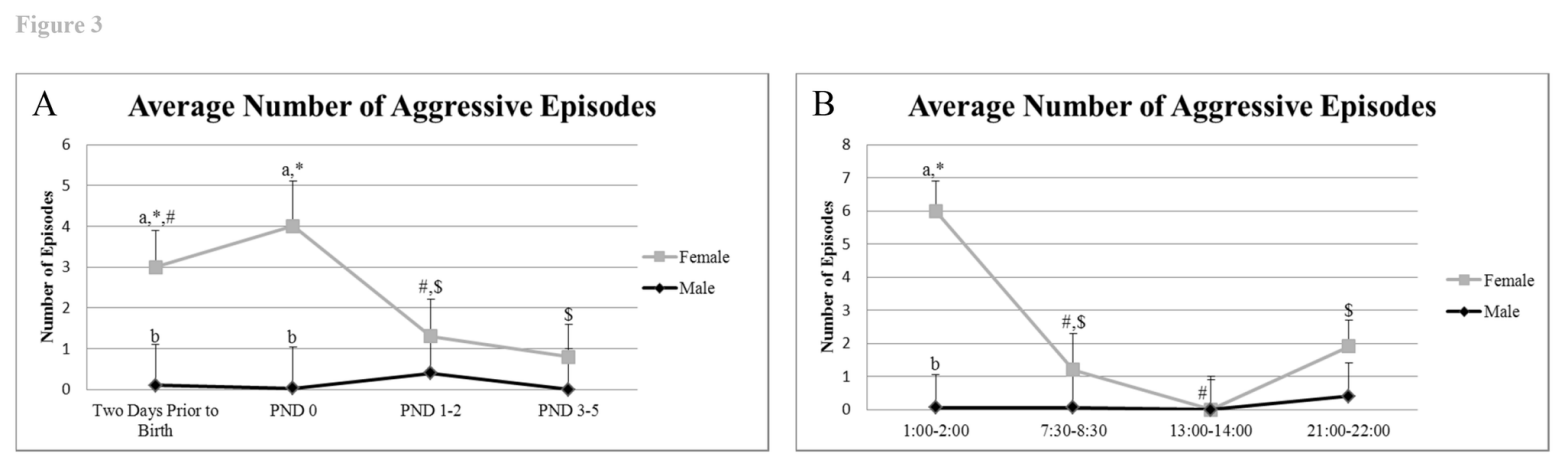
Frequency of aggressive episodes prior to and after birth and throughout the timepoints examined. A) Average number of aggressive episodes across days. B) Average number of aggressive episodes based on time of day. ^*, #, $^ indicates significant differences within sex across days or times examined (P < 0.05). ^a^,^b^ indicates significant differences between sexes on the same day or time examined (P < 0.05).

Females were more aggressive than males two days priors to birth (P = 0.02) and on the day of birth (P = 0.05), but these differences dropped to insignificance subsequently (Figure 3). Throughout, males rarely attacked their partners. These aggressive acts by the female to their male partners occurred primarily in the MD period (Figure 1B) (aggressive acts by female 6.0 ± 1.1 *versus* 0.06 ± 1.1 for male; P = 0.0002) and were barely observed at ML. They were predominantly aggressive in the form of biting and, by the day of birth, these acts were sufficient to drive the male from the nest area for extended periods (Video S1), although the male was able to provide some parental care on this day, as detailed below.

#### Grooming, sniffing and rebreeding partner

**Figure 4:**
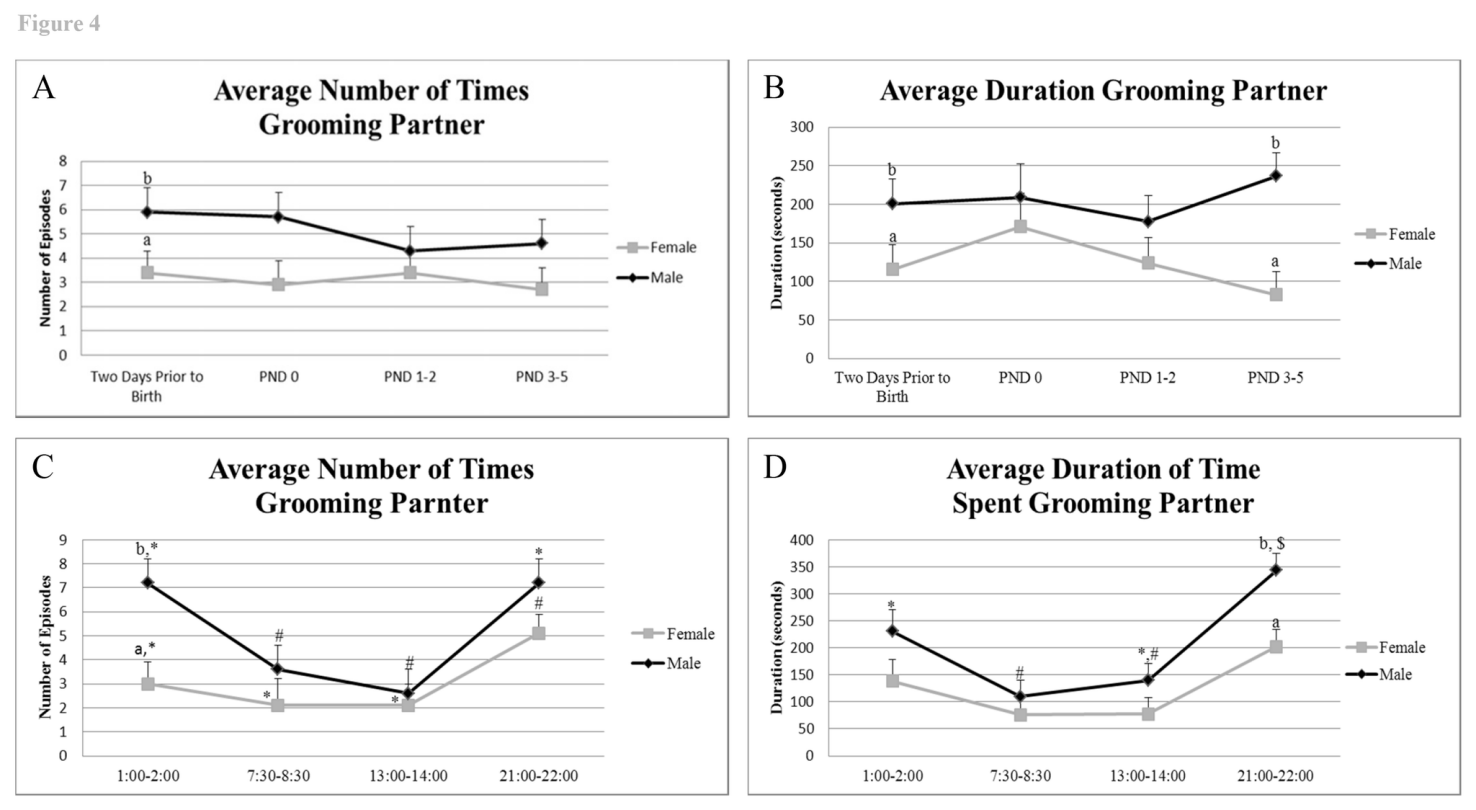
Frequency and duration of partner grooming behaviors prior to and after birth and throughout the timepoints examined. A) Average frequency of grooming partner across trial days. B) Average duration of grooming partner across trial days, C) Average frequency of grooming partner based on time of day, and D) Average duration of grooming partner based on time of day. ^*, #, $^ indicates significant differences within sex across days or times examined (P < 0.05). ^a^,^b^ indicates significant differences between sexes on the same day or time examined (P < 0.05).

While the total number of grooming episodes of their partner did not differ between the sexes (P = 0.09) there was a tendency for males to engage in more of this activity than the females, with the difference being significant in the two-day period before birth of the pups (Figure 4A and B, Videos S1 and S2) (P = 0.05). Males groomed the females for longer than they themselves were groomed (205.9 sec versus 123.2 sec; P = 0.009). Further examination revealed that two days prior to birth, the male groomed his partner more frequently than she did him (male grooming female= 5.7 ± 1.0, female grooming male= 3.4 ± 0.9) (Figure 4A). This activity by the male towards his partner was more frequent and of longer duration during the late light/ night period (LL and MD) (Figure 4C & D).

The number of times the males and females sniffed their partner and the number of times the male attempted to rebreed his female partner were also determined (Figure S2). Males sniffed their partners more frequently prior to birth, on the day of birth, and on PND 1-2 than on PND 3-5) (P value range = 0.001 to 0.04) (Figure S2). Most of this activity occurred during MD (P < 0.0001) (Figure S2). Attempts of the male to mount the female were almost entirely limited to the day of birth (Figure S2) (P < 0.05), when the female was at her most aggressive (Figure 3A).

### Maternal and paternal behaviors

#### Grooming and sniffing pups

**Figure 5:**
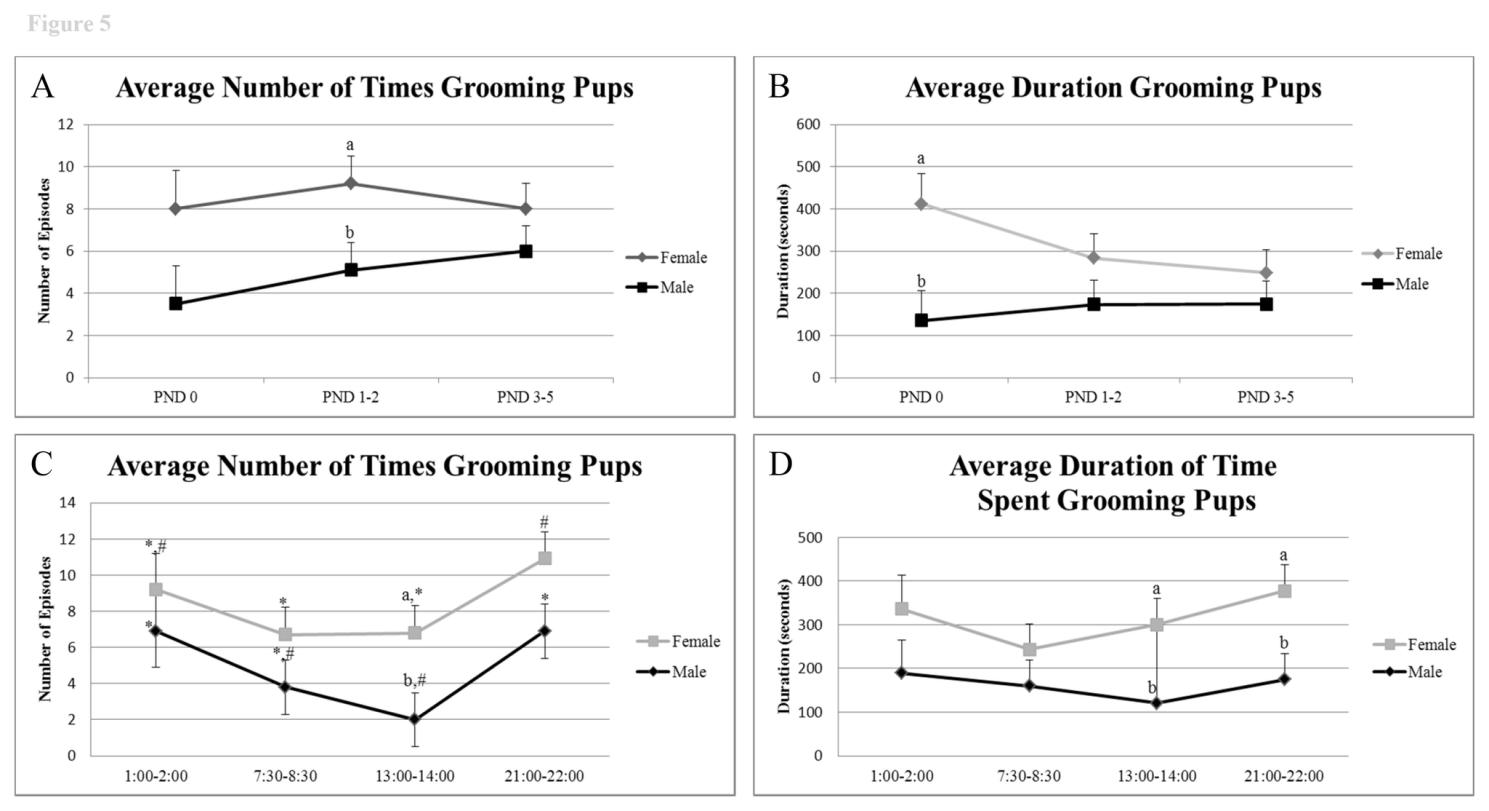
Frequency and duration of grooming pups from PND 0 to 5 and throughout the timepoints examined. A) Average frequency of grooming pups across trial days. B) Average duration of grooming pups across trial days, C) Average frequency of grooming pups based on time of day, and D) Average duration of grooming pups based on time of day. ^*, #^ indicates significant differences within sex across days or times examined (P < 0.05). ^a^,^b^ indicates significant differences between sexes on the same day or time examined (P < 0.05).

The female groomed and licked the pups from PND 0 to 5 almost twice as frequently as did the males (n= 8.4 ± 0.9 versus 4.9 ± 0.9, respectively, P = 0.02), but this difference had largely disappeared by PND 3-5 with males also demonstrating marked grooming episodes and duration of grooming and licking the pups) (Figure 5, Video S2). Average duration of grooming and licking also narrowed between the partners after PND 0, although the total number of such episodes decreased (Figure 5B). As expected, females exhibited greater overall duration than the males in licking and grooming the pups over PND 0 to PND 5 (314.2 ± 40.2 seconds versus 160.9 ± 40.2 seconds; P= 0.02). These aspects of offspring care were spread somewhat evenly throughout the 24 h light/dark period, but tended to be highest during the night (Figure 5C & D). Female involvement was lower in the EL and ML than MD (P value range = 0.02 to 0.04 for frequency).

The overall percentage of time the dams devoted to grooming the pups was about twice that of the fathers (8.8% *versus* 4.5%, P= 0.02). This difference was predominantly due to PND 0 where the females groomed the pups 11.4% of time while the males devoted only 3.7% of their activity to this task (P = 0.01), presumably because he was largely prevented from accessing the pups. The percentage of time spent grooming did not differ significantly between the females and males at any other day of assessment.

While there were no overall sex differences in sniffing pups across the trials (P= 0.1), there were variations between the sexes across days and over the light/dark period emerged (Figure S3). On PND 3-5, the dams sniffed their pups more frequently than the fathers (P = 0.004). Sniffing by the females also increased over PND (P = 0.001) (Figure S3), and this activity by both sexes was predominantly in the MD period (females, P < 0.0001; males, P = 0.05) (Figure S3).

#### Time spent in the nest

**Figure 6:**
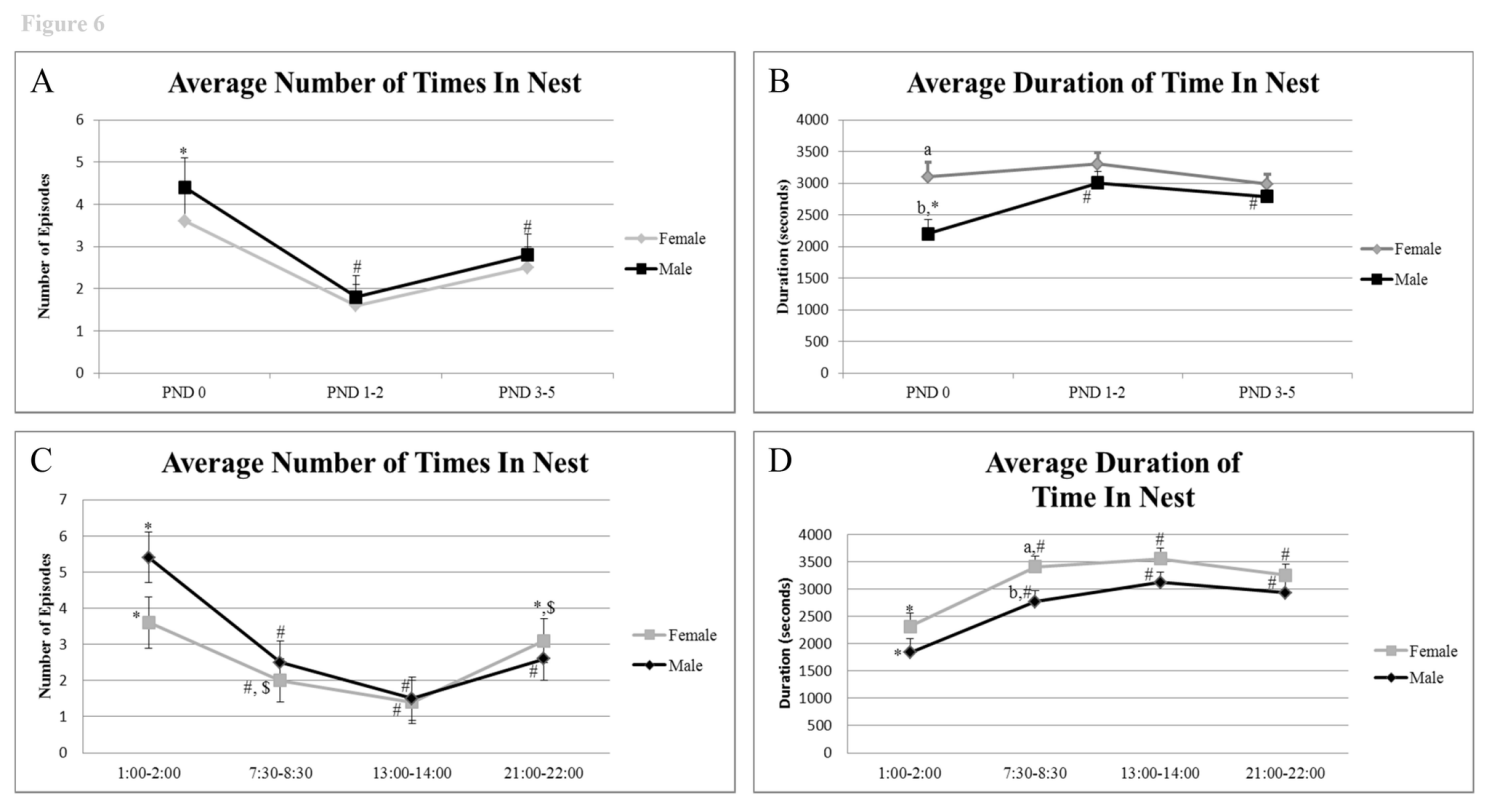
Frequency and duration of time in nest with pups from PND 0-5 and throughout the timepoints examined. A) Average frequency of time in nest across trial days. B) Average duration of time in nest across trial days, C) Average frequency of time in nest based on time of day, and D) Average duration of time in nest based on time of day. ^*, #, $^ indicates significant differences within sex across days or times examined (P < 0.05). ^a^,^b^ indicates significant differences between sexes on the same day or time examined (P < 0.05).

Both males and females tended to move in and out of the nest throughout the trial period and 24 light/dark cycle and to remain with the pups for periods rarely extending beyond 1 h but rarely less than 30 min (Figure 6). There were no overall differences over the entire trial period in the number of times either the mother or father entered the nest (P = 0.5). Attempted entries by males were highest on PND 0, but declined over PND 1-2 and PND 3-5 (P value range 0.001 to 0.03) (Figure 6). These entries were most frequent during the MD period (Figure 6). Female entries were lowest at PND 1-2 and, like the male, were more frequent during the night hours (Figure 6).

The dams exhibited greater overall duration of time in the nest from PND 0-5 over the 3,600 sec observation period than the fathers (3128. 2 ± 132.0 *versus* 2664.0 ± 131.6, P= 0.03), in large part attributable to the differences observed on PND 0 (3100.7 ± 224.0 seconds *versus* 2200.7 ± 221.2 seconds, P = 0.01) (Figure 6B). The total average duration values for time spent in the nest per day calculated on the basis of the four observation periods (Figure 6D) were as follows: PND 0, females, 20.67 h, males 14.7 h, PND 1-2, females 22 h, males 20 h; PND 3-5, females 19.9 h, males,18.6 h.

For each of the one hour assessments from PND 0-5, the dams spent a greater percentage of time in the nest than the males (86.9% versus 74.0%, respectively, P < 0.03). This difference was largely attributable to the greater percentage of time the females spent in the nest on PND 0 compared to the males (86.1% versus 61.1%, P = 0.001). There was no difference between the parents on the other days. Parents spent the least percentage of time in the nest during the MD period compared to the other three time periods (P value range < 0.0001 to 0.001).

#### Time spent nursing by females

**Figure 7:**
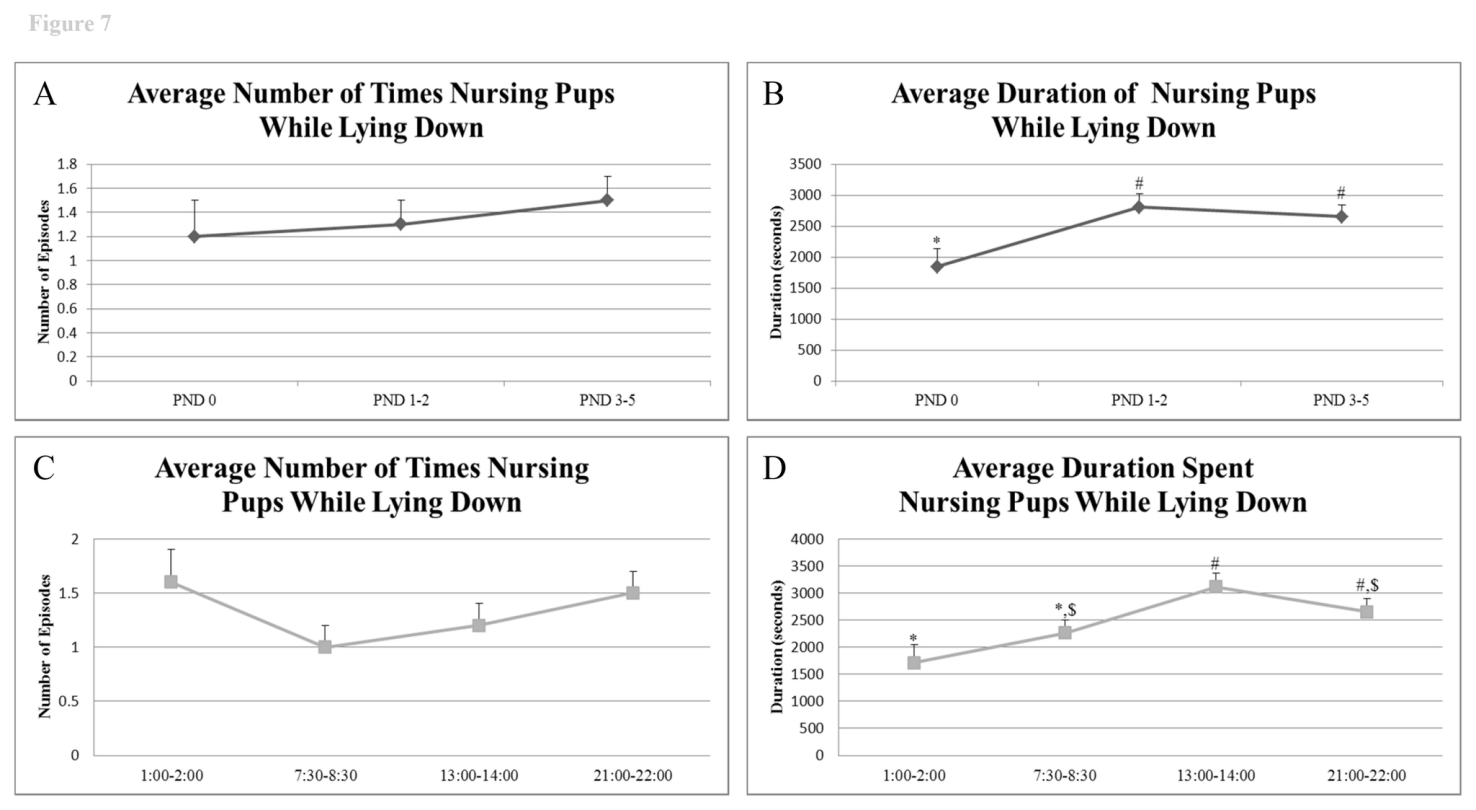
Frequency and duration of time nursing from PND 0-5 and throughout the timepoints examined. A) Average frequency of nursing across trial days. B) Average duration of nursing across trial days, C) Average frequency of time nursing based on time of day, and D) Average duration of time nursing based on time of day. ^*, #, $^ indicates significant differences for females across days or times examined (P < 0.05).

The females spent the majority of their time when they occupied the nest nursing their pups (Figure 7). No differences were observed in the total number of nursing episodes over the PND 0-5 period (Figure 7) (P = 0.1). However, the mean duration of time the female spent nursing increased from PND 0 to PND 5 (P value range 0.009 to 0.02) (Figure 7B). Calculated duration of nursing per day was 12.3 h, 18.7 h, and 17.7 h on PND 0, PND 1-2, and PND 3-5, respectively. While there was no difference in the number of nursing episodes based on time of day, the duration of nursing was affected (P= 0.007) (Figure 7C and D). Nursing was at its lowest frequency in the MD period compared to the other observation periods (P value range 0.001 to 0.02).

### Combined biparental results

#### Grooming pups

To determine the combined parental investment in grooming pups, the average total sum of time that both parents groomed the pups together was determined (Figure S4). The combined grooming episodes were greatest on PND 1-2 and PND 3-5 compared to PND 0 (P value range, 0.0002 to 0.03) (Figure S4). The average total duration that both parents groomed the pups increased as the pups matured (Figure S4). For example, the episodes lasted significantly longer during PND 3-5 compared to PND 0 (P= 0.008). Both parents engaged in more grooming episodes and duration during MD than ML (P = 0.02), although differences between all other periods were not significant (Figure S4).

#### Time spent in nest with pups

The number of occasions where one or both parents huddled over the pups was greater on PND 1-2 and PND 3-5 relative to PND 0 (P value range= 0.0003 to 0.002) (Figure S5). Both parents occupied the nest together for longer times on PND 3-5 than on PND 0 and PND 1-2 (P value range <0.0001 to 0.005). Co-occupation of the nest by the parents was also greatest during the LL period relative to the other three periods in which measurements were made (P value range, 0.003 to 0.04), but the both parents were more frequently in and out of the nest during the MD period (P value range= 0.003 to 0.04) (Figure S5)

#### Pup body weight and temperature, nest temperature, and parent body temperatures

There were no sex differences in trajectory of pup body weight growth from PND 2 to 20 and on the day prior to and after weaning (Figure S6, P = 0.2). From PND 2-18, the pups weighed more each day than on the prior assessment day (P value range < 0.0001 to 0.05), i.e. they grew steadily. Predictably from PND 20 to PND 30, the pups also gained weight (P < 0.0001), but no weight gain was noted in either sex on the day after weaning (P = 0.2).

**Figure 8:**
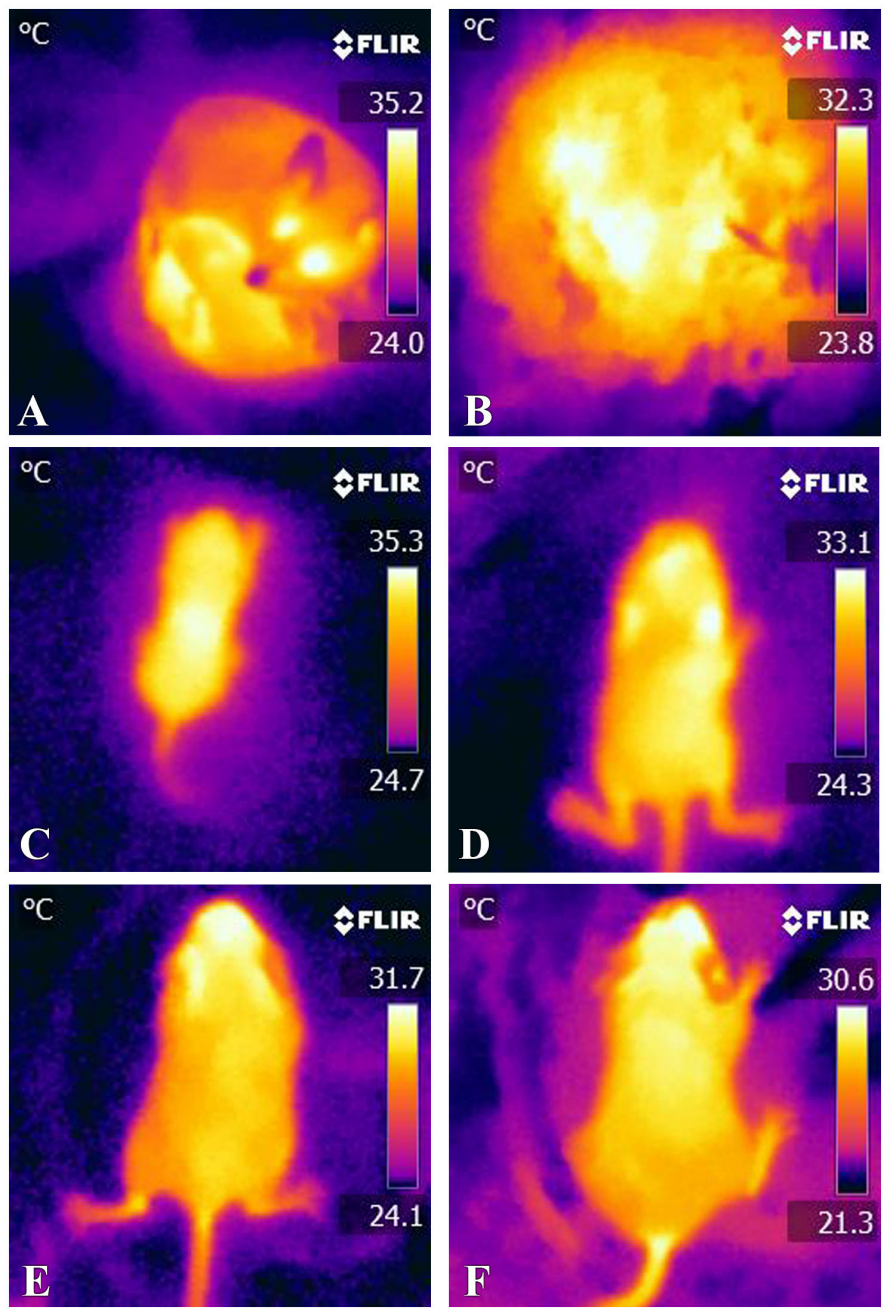
Example thermal images of pups and nest. A. Newborn (PND 2) pups latched on and suckling from dam. Thermal imaging analysis with increased degree of white correlating with increased heat reveals that the exothermic pups exhibit at this age a higher body temperature than the dam. B. Thermal image of nest temperature that was measured to determine the impact of biparental care. Thermal imaging analysis of representative pups at PND 2 (C), PND 10 (D), PND 20 (E), and PND 30 (prior to weaning, F). Panels C-F reveal that the pup body temperature decreases significantly from PND 2 to PND 20 (P < 0.0001).

**Figure 9:**
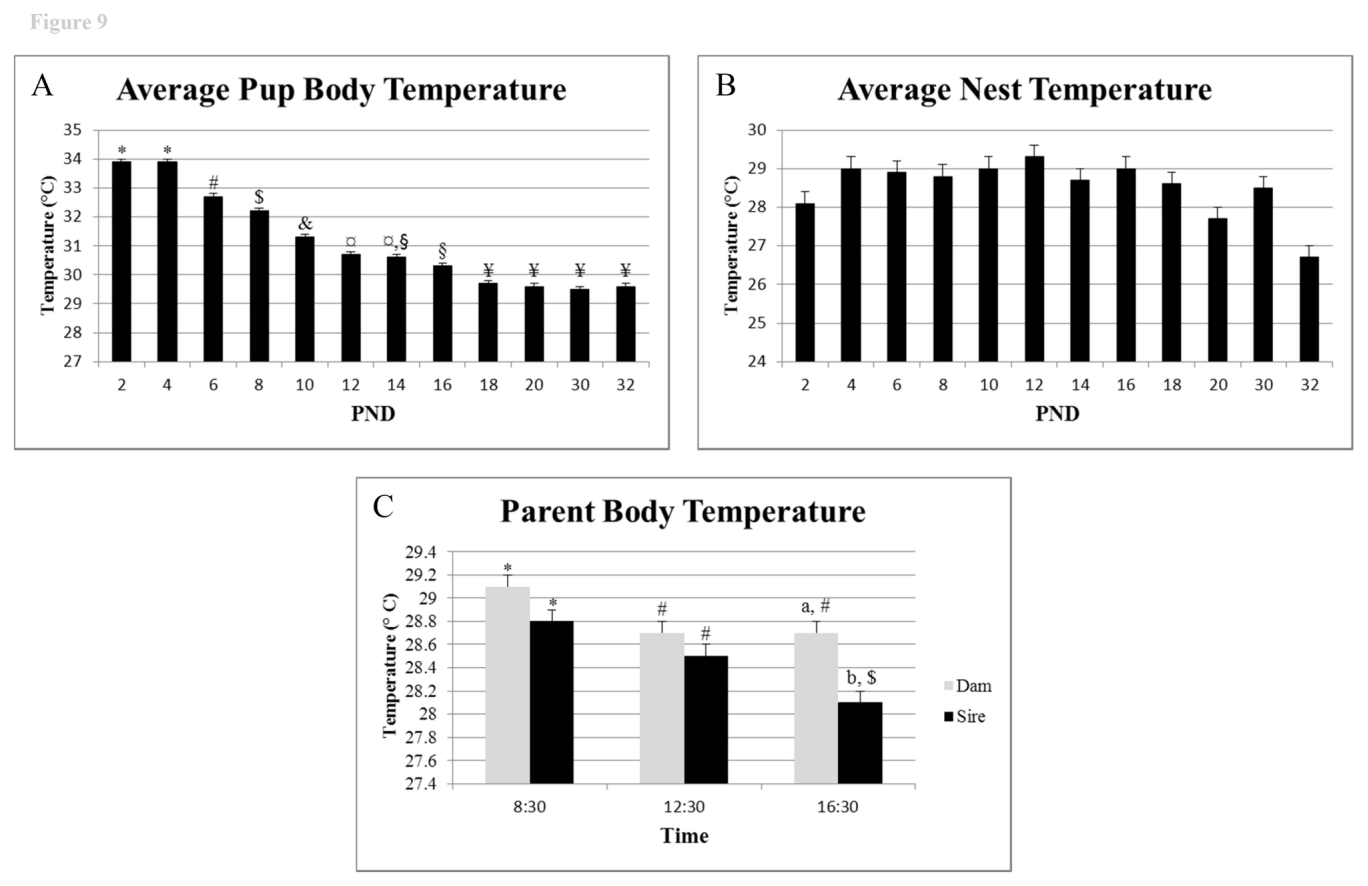
Pup, nest, and parent body temperature. A) Average pup body temperature across days. B) Average nest temperature across days. C) Average parent body temperature based on time of day. ^*, #, $, &, °, §, ¥^ indicates significant differences in temperature across days or times examined for pups (A) or between parents (C) (P < 0.05).

Pup body temperature decreased from PND 2 (33.9 °C) to PND 32 (29.6 °C, P < 0.001, Figures 8 and 9). By PND 18, body temperatures (29.8 °C) stabilized and remained constant until PND 30 (just prior to weaning) and PND 32 (post-weaning). No clear circadian patterns of pup body temperature emerged over the course of the study. Nest temperature remained fairly constant from PND 2 until PND 18 (Figure 8) and did not change significantly over the light cycle (data not shown).

Dams exhibited a slightly higher body temperatures than their male partners (28.8 °C compared to 28.4 °C, P = 0.0001). In both sexes, body temperature varied throughout the day (P < 0.0001), with values highest in the morning compared to mid and late- afternoon (P value range < 0.0001 to 0.04) (Figure 9).

## Discussion

The primary focus of the current work was aimed at assessing a readily measurable complement of biparental behaviors in California mice that occur just prior to and in the following days after the mother delivers her litter of pups. By tracking the behaviors at selected times throughout the dark and light cycles for the same pairs, circadian patterns of behavior could be assessed for both partners. Together they provide insights into the social interactions that occur between bonded male and female California mice and between them and their pups.

Not unexpectedly for a nocturnal species, both the males and females tended to be more active during the middle of the night or dark cycle (MD period) as evidenced by increased eating and drinking, nursing by the female, self-grooming, movement in and out of the nest, and interactions between the pair bond and pups (grooming and sniffing episodes). While there was no difference in eating habits displayed by the males before and after birth, females ate more often and for longer periods over the two days preceding birth than at other times, presumably in preparation for nursing and delivery of the pups. On the day of birth, however, the females redirected much more of their time to attending to the pups, including nursing and grooming, and reduced the number and duration of eating episodes, although feeding partially rebounded from PND 1-5, when the males began to share in parental care. The increasing role of the male is also reflected by the reduced amount of time he spent eating and self-grooming during PND 1-5 than before the birth. Together, these observations support the notion that monogamy and biparental care may have evolved in California mice and other monogamous rodents in response to resource availability and the requirement of females to leave the nest and forage to maintain energy reserves for lactation [31,32,33,34,35,42,52,53], while the males in turn attend the pups.

The experiments also confirmed the high degree of male participation in parental care in *P* *. californicus* , especially in licking and grooming the pups when the female was present in the nest and in huddling over them when she was absent. Our data are consistent with studies performed with other biparental rodent species that indicate that mothers spend more time grooming the pups than the fathers [54,55,56,57,58].

The use of infra-red video documentation throughout the dark and light cycles did reveal a number of unanticipated behaviors. For example, the females exhibited aggressive episodes towards their partners in the days leading up to and on the day of the birth, particularly during the dark cycle when both sexes were most active. In no case did the male retaliate but instead would usually leave the nest area, especially on PND 0 (Figure 3, Video S1). The frequent attempts of the males to copulate with their female partners on PND 0 were also usually rebuffed, but must on occasions have been successful as nearly all the females birthed a second litter soon after the first had been weaned (data not shown). Interestingly, the length of gestation (~30 days) is similar to the length of the weaning period in this species [29,43]. To our knowledge, these data provide the first evidence of female *P* *. californicus* demonstrating aggression to her pair-bonded mate during the period leading up to the birth of pups, but somewhat similar findings have been reported for the southern grasshopper mouse ( *Onychomys* *torridus* ) where the father is excluded from the nest by the female on the first few days post-partum [56] and in monogamous, biparental cichlid fish ( *Amatitlanianigrofasciata* ) [59]. Male Mongolian gerbil (*Meriones unguiculatus*) fathers also avoid the nest site for several hours after parturition, but this response appears to be due to lack of prior experience rather than to active exclusion by their female partners [60]. In the California mouse, the aggressive acts by the female may serve to prevent the male engaging in infanticide on the day of birth, but also to prime her partner for increased parental responsibilities over the days that follow [25].

Rather than aggression, the male California mouse demonstrates several affiliative behaviors towards his partner, as evidenced by the frequency and duration of time spent grooming and sniffing the female, particularly during the two days prior to birth (Figure 4, Figure S2, Videos S1 and S2). It seems possible that during the period before birth, the female displays chemosensory cues signaling the pending parturition and possibly sexual receptivity, although it was only on PND 0 that the males attempted to rebreed their partners.

Both parents spent a significant amount of their time, often together, in grooming their pups, and it must be assumed that these activities are linked to the survival and future welfare of the offspring. Such grooming presumably benefits the pups in the short term by removing fetal membranes and enhancing peripheral blood circulation [61], activating suckling behavior [62], stimulating reflexive urination [63] and cleansing the body to reduce the spread of disease and the attention of predators in the wild [64]. There may also be longer term benefits. The extent of maternal anogenital licking of young may affect later sexual development of male rats and lead to epigenetic changes in mice that have been linked to adult behaviors [16,65,66,67,68,69,70]. It will be of interest to determine whether reduced grooming by one or both parents has adverse outcomes on California mouse young. It has been recently shown in this species that paternal behaviors can be trans-generationally transmitted via epigenetic mechanisms [71].

In Mongolian gerbils, males that possess low testosterone concentrations or that gestated between two females (2F males) *in utero* spend more time in contact with and huddling over their pups than males with high testosterone concentrations or those that developed between two males (2M males) [72,73,74,75,76]. As California mice usually birth an average of 2 pups per litter [29,43,44,45,46], it is difficult to assess the effects of gestation position on later paternal behaviors. Also, in contrast to Mongolian gerbils, California mice fathers with high concentrations of testosterone prove to be better fathers than those of low testosterone status, possibly due to aromatization of testosterone to estradiol in the brain [77]. Furthermore, high testosterone concentrations in male California mice during courtship may be a reliable predictor of later paternal behaviors, including increased huddling over the pups [78]. Finally, high testosterone concentration are positively associated with paternal behaviors, such as huddling over the pups, in the Volcano mouse ( *Neotomodon* *alstoni* ) [79]. The collective studies suggest that the effects of testosterone on paternal behaviors may be species dependent.

California mice pups are exothermic until about half way through the suckling period [39], yet they maintain a body temperature higher than that of their parents until about PND 16, i.e. throughout the period that they are believed to be exothermic. As male and female pups had similar body weights, they would presumably experience similar susceptibilities to excessive body heat loss during their first two weeks or so of development. Moreover, litter size is small (usually ~ 2 pups) in *P* *. californicus* [29,43,44,45,46], and so heat generated from the litter may have much less value in maintaining pup body temperature in the unattended nest than in species where the litter is large, as in rabbits and rats [47,80,81]. Even there, position in the neonatal litter huddle affects pup development [82]. As in Djungarian hamsters ( *P* *. campbelli* ) [83], pup well-being in *P* *. californicus* may depend upon the presence of the father, particularly if the mother is foraging and ambient temperatures fall, which is a more prolonged risk than encountered in rats whose pups are able to control their body temperatures as early as one week after birth [84]. The elevated body temperatures of the California mice pups in the early postnatal period is likely due to the near constant attention of one or both parents. In rat pups, at least, warmer conditions in the days following birth have positive benefits, including earlier growth of fur, increased body weight, and earlier nest eggression and weaning [85,86].

In conclusion, the current data expand upon the existing knowledge of biparental care in *P* *. californicus* by examining the individual and social behaviors prior to and after birth. Together, the data provide detailed information on parental care investment by both parents. To our knowledge, this is first study to demonstrate the exclusion of the male from the nest and aggressive acts by the female in the period leading up to the birth and the increasing involvement of the male in subsequent days. The data emphasize the likely importance of licking and grooming, as well as sniffing the young, as vital aspects of the behavior for both parents that likely contribute to successful development of the offspring. These studies also provide direct, quantitative evidence that males make an important contribution to parental care by huddling over the pups while the female is absent from the nest, which may be especially important to maintain thermoregulation, Patterns of eating behavior suggest that biparental care may have evolved, in part, to permit the female time to leave the nest to forage at night and thereby maintain her energy and fat reserves essential for lactation and suckling, consistent with conclusions advanced by others [31,32,33,34,35,42,52,53]. . The current studies are important in that they provide a framework for the normal pair-bond behaviors that occur between California mice, including aggression on the part of the female to her male partner prior to and after birth. Males do not reciprocate these acts, but instead are attentive to their partner in the form grooming and sniffing. Additionally, by understanding the normal suite of parental behaviors and pup parameters, assessments can now be made on how environmental and other factors may disrupt these processes. Such studies are particularly important to understand in males, where so little information is available from other rodent models.

## Supporting Information

Figure S1 **Frequency and duration of eating and drinking throughout the light and dark cycles.** A) Average frequency of eating for both parents based on time of day. B) Average duration of eating for both parents based on time of day. C) Average frequency of drinking for both parents based on time of day. D) Average duration of drinking based on time of day. ^*,#^ indicates significant differences within sex across times examined (P < 0.05). ^a^,^b^ indicates significant differences between sexes at the same time of day (P < 0.05).
(PDF)

Figure S2 **Frequency and duration of select social behaviors between the pair-bond prior to and after birth and throughout the timepoints examined.** A) Average number of times sniffing partner across days. B) Average number of time sniffing partner based on time of day. C) Average number of episodes male observed re-breeding female based on time of day. ^*,#, $^ indicates significant differences within sex across days or times examined (P < 0.05). ^a^,^b^ indicates significant differences between sexes at the same day or time examined (P < 0.05).
(PDF)

Figure S3 **Frequency of sniffing pups from PND 0 to 5 and throughout the timepoints examined.** A) Average number of times sniffing pups across days. B) Average number of time sniffing pups based on time of day. ^*,#^ indicates significant differences within sex across days or times examined (P < 0.05). ^a^,^b^ indicates significant differences between sexes at the same day or time examined (P < 0.05).
(PDF)

Figure S4 **Combined frequency and duration of time both parents spent grooming pups from PND 0-5 and throughout the timepoints examined.** A) Average combined episodes both parents spent grooming pups across trial days. B) Average combined duration of time both parents spent grooming pups across trial days, C) Average combined episodes both parents spent grooming pups based on time of day, and D) Average combined duration both parents spent grooming pups based on time of day. ^*, #^ indicates significant differences across days or times examined (P < 0.05).
(PDF)

Figure S5 **Combined frequency and duration of time both parents spent in nest with pups from PND 0-5 and throughout the timepoints examined.** A) Average combined episodes parents spent in nest across trial days. B) Average combined duration of time both parents spent in nest across trial days, C) Average combined episodes parents spent in and out of the nest based on time of day, and D) Average combined duration of time both parents spent of in nest based on time of day. ^*, #, $^ indicates significant differences across days or times examined (P < 0.05).
(PDF)

Figure S6 **Male and female pup body weight growth.** Both male and female California mice pups grew at equivalent rates across days.
(PDF)

Table S1 (DOCX)

Video S1 **Demonstration of female aggression to male on the day of birth (PND 0) (in viewer’s lower left hand box).** The male (shaved back) is attempting to be affectionate with the female and approaches the nest. However, she drives him away from the nest by attempting to bite him. Example of partner grooming (viewer’s lower right hand box). Male (shaved back) is grooming the female. Overall, the males groomed their female partners more than they did them.
(WMV)

Video S2 **Example of male parenting in the form of grooming and licking the pup(s).** When the video begins (viewer’s lower left hand box), the pup is latched onto one of the four mammary glands that California female mice possess. Once the pup detaches from the female, the male parent begins to lick both the anogenital and non- anogenital region of the pup. Another example of partner grooming (viewer’s upper right hand box). Again, the male (shaved back) is observed grooming his female partner.
(WMV)
