# Supplementary material for: Interactions between Parents and Parents and Pups in the Monogamous California Mouse (Peromyscus californicus)
Source: PLoS One. 2013 Sep 19;8(9):e75725. doi: 10.1371/journal.pone.0075725 (PMC3777941; doi:10.1371/journal.pone.0075725)
Supplement: Figure S1 — Frequency and duration of eating and drinking throughout the light and dark cycles. A) Average frequency of eating for both parents based on time of day. B) Average duration of eating for both parents based on time of day. C) Average frequency of drinking for both parents based on time of day. D) Average duration of drinking based on time of day. *,# indicates significant differences within sex across times examined (P < 0.05). a,b indicates significant differences between sexes at the same time of day (P < 0.05). (PDF) [file pone.0075725.s001.pdf]

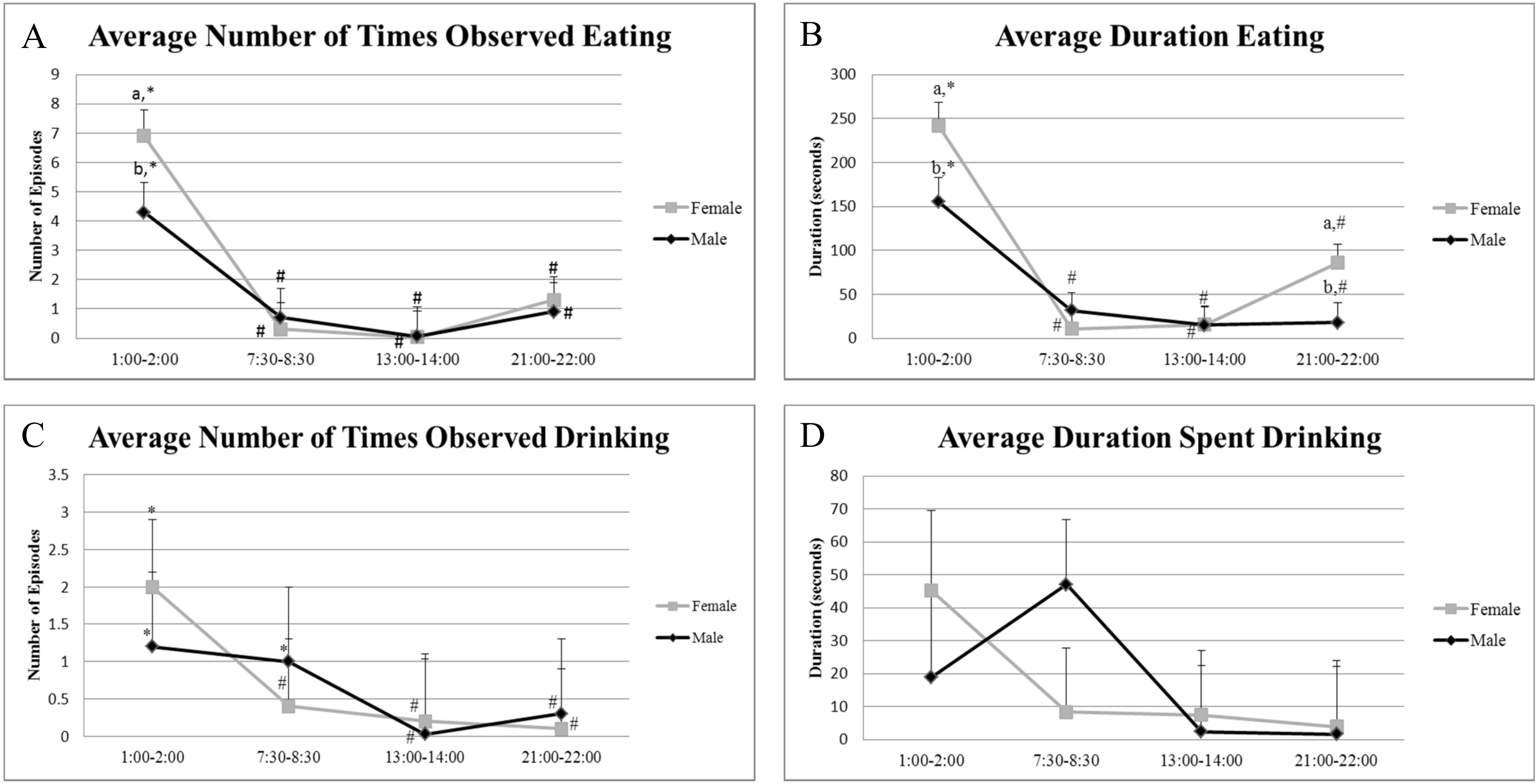
A
Average Number of Times Observed Eating
Number of Episodes
9
8
7
6
5
4
3
2
1
0
a,*
b,*
#
Female
Male
1:00-2:00
7:30-8:30
13:00-14:00
21:00-22:00
B
Average Duration Eating
Duration (seconds)
300
250
200
150
100
50
0
a,*
b,*
a,#
b,#
#
Female
Male
1:00-2:00
7:30-8:30
13:00-14:00
21:00-22:00
C
Average Number of Times Observed Drinking
Number of Episodes
3.5
3
2.5
2
1.5
1
0.5
0
*
#
Female
Male
1:00-2:00
7:30-8:30
13:00-14:00
21:00-22:00
D
Average Duration Spent Drinking
Duration (seconds)
80
70
60
50
40
30
20
10
0
Female
Male
1:00-2:00
7:30-8:30
13:00-14:00
21:00-22:00
