# Supplementary material for: Interactions between Parents and Parents and Pups in the Monogamous California Mouse (Peromyscus californicus)
Source: PLoS One. 2013 Sep 19;8(9):e75725. doi: 10.1371/journal.pone.0075725 (PMC3777941; doi:10.1371/journal.pone.0075725)
Supplement: Figure S2 — Frequency and duration of select social behaviors between the pair-bond prior to and after birth and throughout the timepoints examined. A) Average number of times sniffing partner across days. B) Average number of time sniffing partner based on time of day. C) Average number of episodes male observed re-breeding female based on time of day. *,#, $ indicates significant differences within sex across days or times examined (P < 0.05). a,b indicates significant differences between sexes at the same day or time examined (P < 0.05). (PDF) [file pone.0075725.s002.pdf]

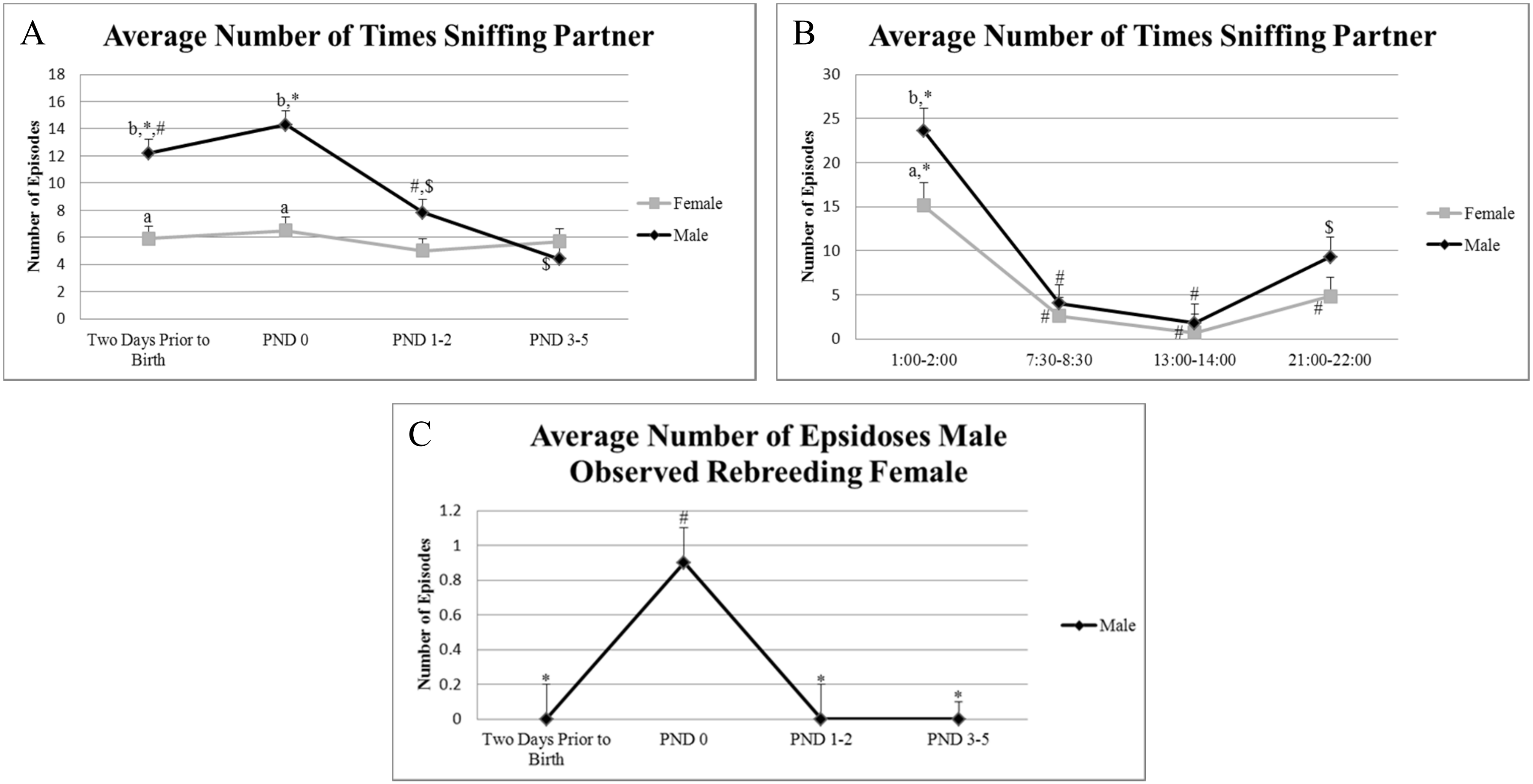
A
Average Number of Times Sniffing Partner
Number of Episodes
18
16
14
12
10
8
6
4
2
0
Two Days Prior to Birth
PND 0
PND 1-2
PND 3-5
b,*,#
b,*
#,$
a
a
$
Female
Male
B
Average Number of Times Sniffing Partner
Number of Episodes
30
25
20
15
10
5
0
1:00-2:00
7:30-8:30
13:00-14:00
21:00-22:00
b,*
a,*
#
#
#
#
$
#
Female
Male
C
Average Number of Epsidoses Male Observed Rebreeding Female
Number of Episodes
1.2
1
0.8
0.6
0.4
0.2
0
Two Days Prior to Birth
PND 0
PND 1-2
PND 3-5
#
*
*
*
Male
