# Supplementary figures and images for: Interactions between Parents and Parents and Pups in the Monogamous California Mouse (Peromyscus californicus)
Source: PLoS One. 2013 Sep 19;8(9):e75725. doi: 10.1371/journal.pone.0075725 (PMC3777941; doi:10.1371/journal.pone.0075725)

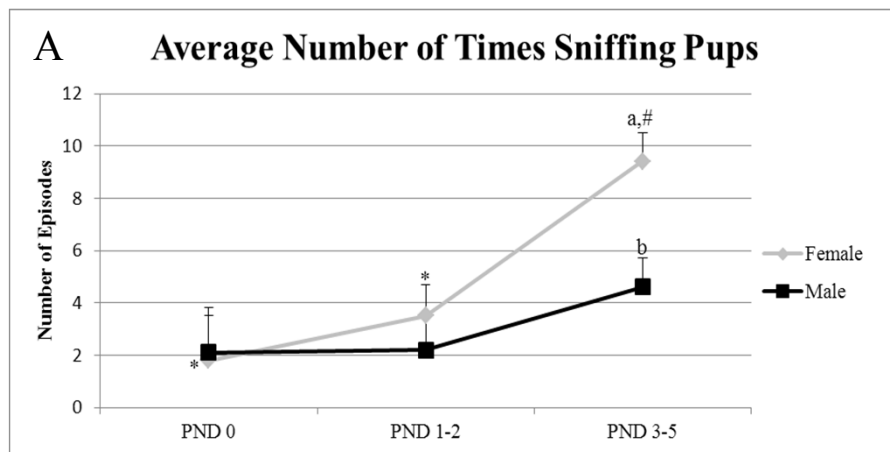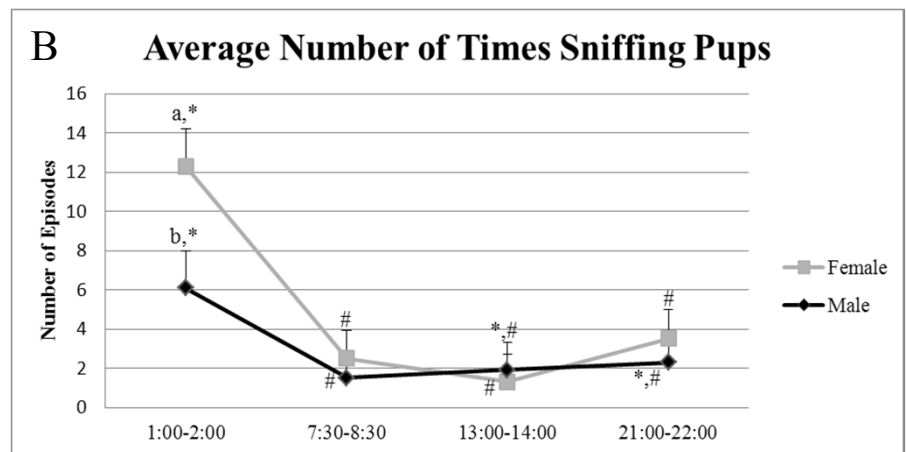

Supplement: Figure S3 — Frequency of sniffing pups from PND 0 to 5 and throughout the timepoints examined. A) Average number of times sniffing pups across days. B) Average number of time sniffing pups based on time of day. *,# indicates significant differences within sex across days or times examined (P < 0.05). a,b indicates significant differences between sexes at the same day or time examined (P < 0.05). (PDF) [file pone.0075725.s003.pdf]

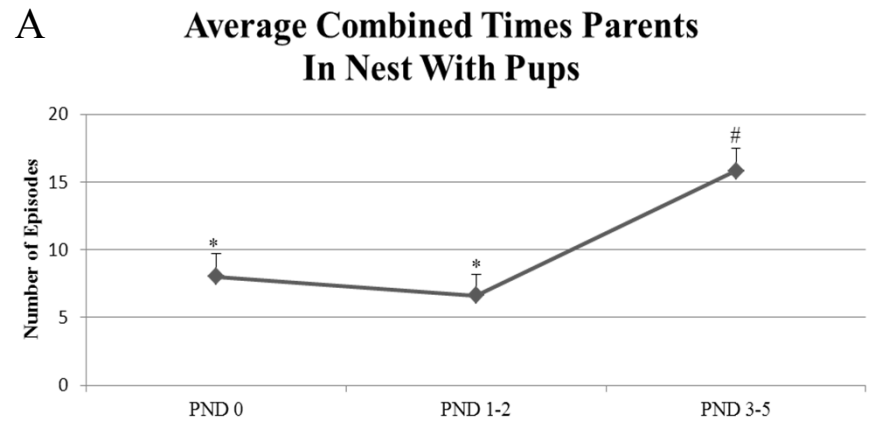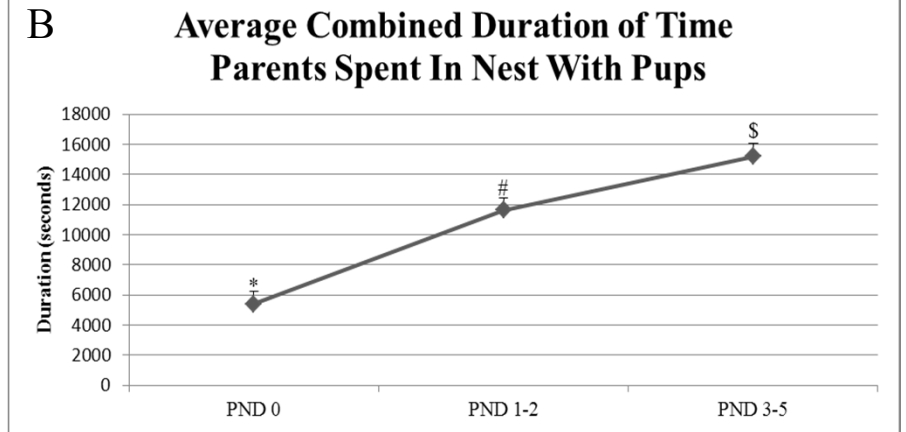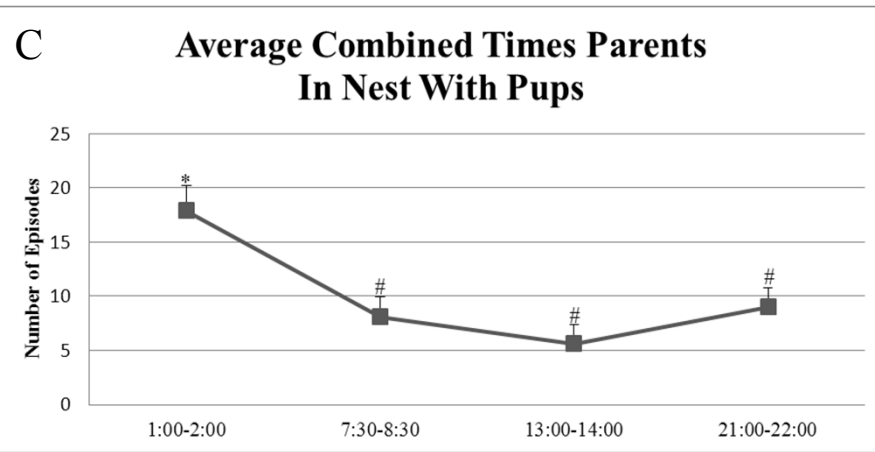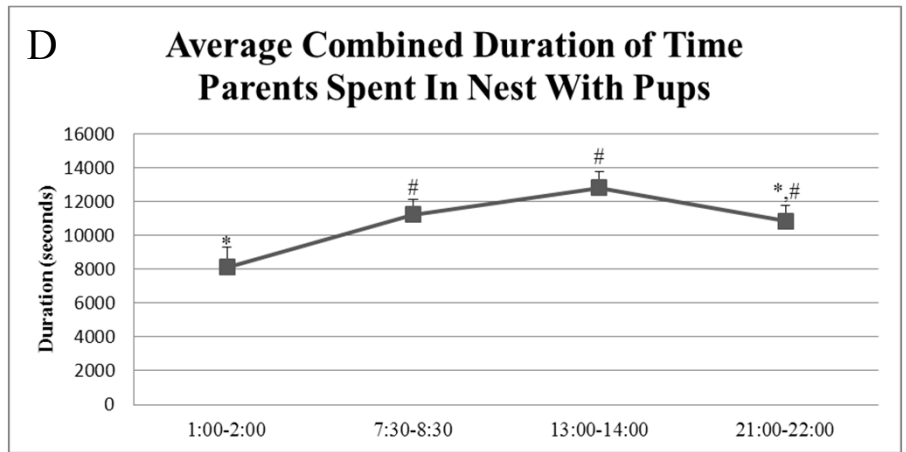

Supplement: Figure S5 — Combined frequency and duration of time both parents spent in nest with pups from PND 0-5 and throughout the timepoints examined. A) Average combined episodes parents spent in nest across trial days. B) Average combined duration of time both parents spent in nest across trial days, C) Average combined episodes parents spent in and out of the nest based on time of day, and D) Average combined duration of time both parents spent of in nest based on time of day. *, #, $ indicates significant differences across days or times examined (P < 0.05). (PDF) [file pone.0075725.s005.pdf]

## Average Pup Body Weight

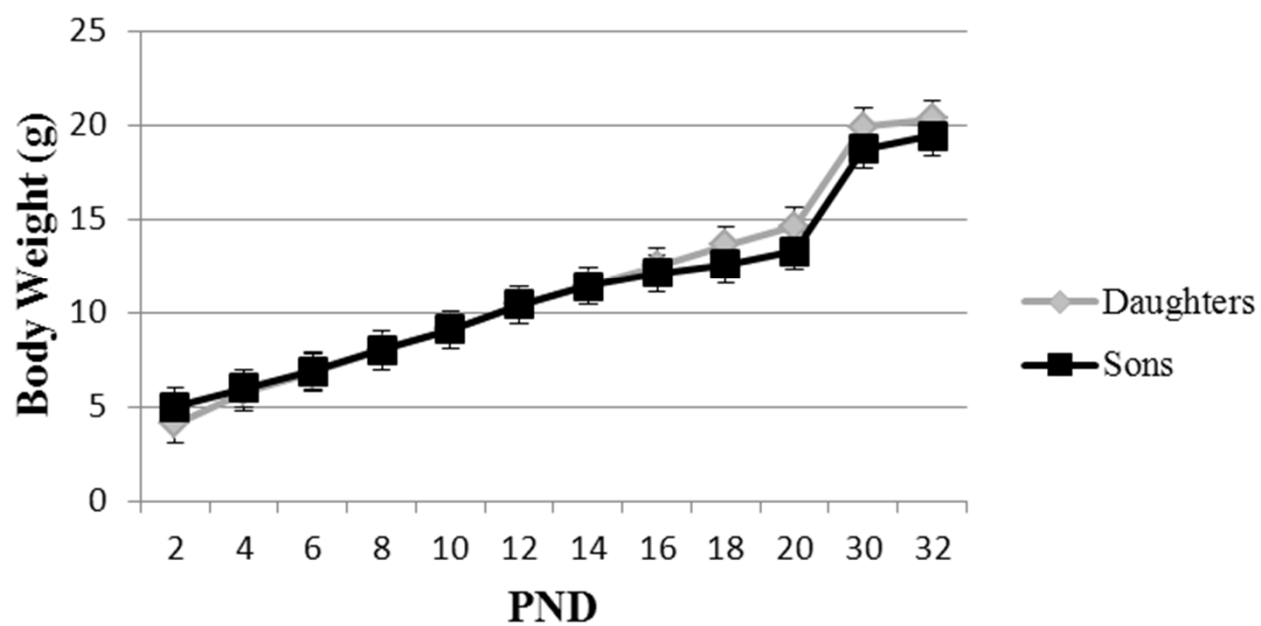

Supplement: Figure S6 — Male and female pup body weight growth. Both male and female California mice pups grew at equivalent rates across days. (PDF) [file pone.0075725.s006.pdf]
