# Supplementary material for: Interactions between Parents and Parents and Pups in the Monogamous California Mouse (Peromyscus californicus)
Source: PLoS One. 2013 Sep 19;8(9):e75725. doi: 10.1371/journal.pone.0075725 (PMC3777941; doi:10.1371/journal.pone.0075725)
Supplement: Figure S4 — Combined frequency and duration of time both parents spent grooming pups from PND 0-5 and throughout the timepoints examined. A) Average combined episodes both parents spent grooming pups across trial days. B) Average combined duration of time both parents spent grooming pups across trial days, C) Average combined episodes both parents spent grooming pups based on time of day, and D) Average combined duration both parents spent grooming pups based on time of day. *, # indicates significant differences across days or times examined (P < 0.05). (PDF) [file pone.0075725.s004.pdf]

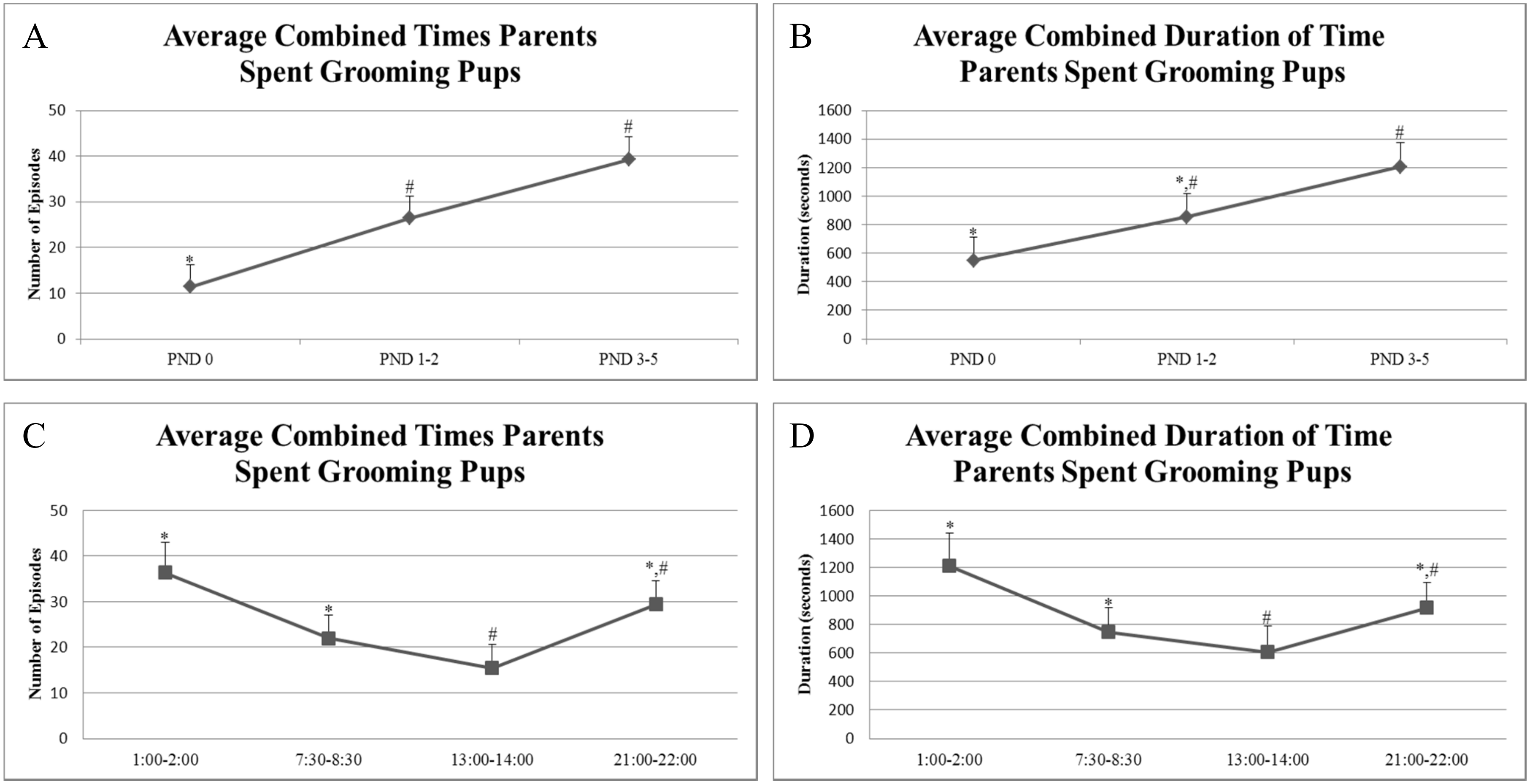

A
Average Combined Times Parents Spent Grooming Pups
Number of Episodes
0
10
20
30
40
50
PND 0
PND 1-2
PND 3-5
*
#
#
B
Average Combined Duration of Time Parents Spent Grooming Pups
Duration (seconds)
0
200
400
600
800
1000
1200
1400
1600
PND 0
PND 1-2
PND 3-5
*
*,#
#
C
Average Combined Times Parents Spent Grooming Pups
Number of Episodes
0
10
20
30
40
50
1:00-2:00
7:30-8:30
13:00-14:00
21:00-22:00
*
*
#
*,#
D
Average Combined Duration of Time Parents Spent Grooming Pups
Duration (seconds)
0
200
400
600
800
1000
1200
1400
1600
1:00-2:00
7:30-8:30
13:00-14:00
21:00-22:00
*
*
#
*,#
