# Supplementary material for: Interactions between Parents and Parents and Pups in the Monogamous California Mouse (Peromyscus californicus)
Source: PLoS One. 2013 Sep 19;8(9):e75725. doi: 10.1371/journal.pone.0075725 (PMC3777941; doi:10.1371/journal.pone.0075725)
Supplement: Table S1 — (DOCX) [file pone.0075725.s007.docx]

| **Table S1:** Individual, social, and parental behaviors that were assessed | | |
| --- | --- | --- |
| **Individual Behaviors** | **Behaviour** | **Operational Definition** |
|  | Eating or drinking | Female or male is eating or drinking (frequency and duration measured) |
|  | Self-grooming | Female or male is self-grooming (frequency and duration measured) |
| **Social Behaviors** | Aggressive to partner | Female or male is aggressive to his/her partner (frequency measured) |
|  | Grooming Partner | Female or male is grooming his/her partner (frequency and duration measured) |
|  | Sniffing partner | Female or male is sniffing his/her partner (frequency measured) |
|  | Rebreeding | Male is rebreeding the female (frequency measured) |
| **Parental Behaviors** | Grooming and licking pups | Female or male licking or grooming pups on any part of the body, including the ano-genital region (frequency and duration measured) |
|  | Sniffing pups | Female or male is sniffing the pups (frequency measured) |
|  | In nest | Female or male is inside the nest and huddling over the pups (frequency and duration measured) |
|  | Nursing and lying down | Female is nursing pups while lying down (frequency and duration measured) |
